# Supplementary material for: Factorial Invariance of the Satisfaction with Life Scale (SWLS) in Mexican and Colombian University Students
Source: Behav Sci (Basel). 2025 Feb 26;15(3):277. doi: 10.3390/bs15030277 (PMC11939420; doi:10.3390/bs15030277)
Supplement: Supplementary file 1 [file behavsci-15-00277-s001.zip › behavsci-3487177-supplementary.pdf]

Material suplementario/ Supplementary Material

Escala de Satisfacción con la Vida (SWLS) utilizada en el estudio actual/ Satisfaction With Life Scale (SWLS) used in the current study.

Instrucciones:

A continuación, encontrarás cinco afirmaciones con las que puedes estar de acuerdo o en desacuerdo. Indica tu grado de acuerdo con cada afirmación:

		Completamente en desacuerdo	Bastante en desacuerdo	Un poco desacuerdo	Un poco de acuerdo	Bastante de acuerdo	Completamente de acuerdo
1	En muchos aspectos, tu vida se acerca a su ideal	☐	☐	☐	☐	☐	☐
2	Tus condiciones de vida son excelentes	☐	☐	☐	☐	☐	☐
3	Estás satisfecho/a con tu vida	☐	☐	☐	☐	☐	☐
4	Hasta ahora has obtenido las cosas importantes que has deseado en tu vida	☐	☐	☐	☐	☐	☐
5	Si pudieras vivir tu vida de nuevo, no cambiarías nada	☐	☐	☐	☐	☐	☐

The Satisfaction With Life Scale (Dienner, 1985).

Instructions for administering the scale are: Below are five statements with which you may agree or disagree. Using the 1-7 scale below, indicate your agreement with each item by placing the appropriate number on the line preceding that item. Please be open and honest in your responding. The 7-point scale is: 1 =strongly disagree, 2 = disagree, 3 = slightly disagree, 4 = neither agree nor disagree, 5 =slightly agree, 6 =agree, 7 =strongly agree.

		strongly disagree	disagree	slightly disagree	neither agree nor disagree	slightly agree	agree	strongly agree.
1	In most ways my life is close to my ideal.	☐	☐	☐	☐	☐	☐	☐
2	The conditions of my life are excellent.	☐	☐	☐	☐	☐	☐	☐
3	I am satisfied with my life	☐	☐	☐	☐	☐	☐	☐
4	So far I have gotten the important things I want inlife.	☐	☐	☐	☐	☐	☐	☐
5	If I could live my life over, I would change almost nothing.	☐	☐	☐	☐	☐	☐	☐
